# Spectral analysis of amplitude and phase echoes in picosecond ultrasonics for strain pulse shape determination

**DOI:** 10.1016/j.pacs.2023.100566

**Published:** 2023-10-29

**Authors:** Takehiro Tachizaki, Jeremy J. Baumberg, Osamu Matsuda, Motonobu Tomoda, Hirotsugu Ogi, Oliver B. Wright

**Affiliations:** a School of Information Science and Technology, Tokai University, Hiratsuka, Kanagawa, 259-1292, Japan; b NanoPhotonics Centre, Cavendish Laboratory, University of Cambridge, Cambridge, CB3 0HE, United Kingdom; c Faculty of Engineering, Hokkaido University, Sapporo, 060-8628, Japan; d Graduate School of Engineering, Osaka University, Yamadaoka 2-1, Suita, Osaka, 565-0871, Japan; e Hokkaido University, Sapporo, 060-0808, Japan

**Keywords:** Picosecond ultrasonics, Strain pulse, Photoelastic effect, Optical interferometry, Ultrafast

## Abstract

We introduce a spectral analysis method in picosecond ultrasonics to derive strain pulse shapes in a opaque sample with known optical properties. The method makes use of both the amplitude and phase of optical transient relative reflectance changes obtained, for example, by interferometry. We demonstrate this method through numerical simulation and by analysis of experimental results for a chromium film.

## Highlights

• We extract the strain pulse shape in picosecond ultrasonics with an analytical method.
• The method relies on the Fourier processing of echoes using a filter function.
• We validate the method with experimental results for ultrafast reflectance changes.

## Graphical abstract

**Figure:**
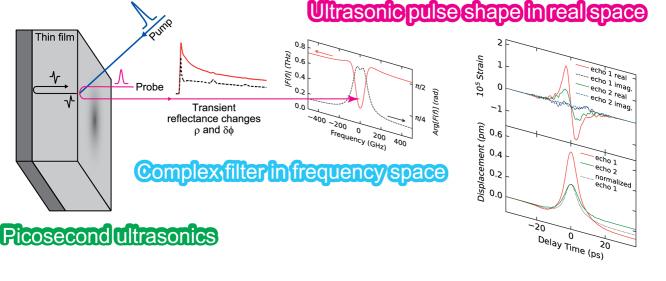

## 1 Introduction

The advent of ultrashort light pulses made possible the generation and detection of picosecond strain pulses by a non-contact and nondestructive technique known as picosecond laser ultrasonics or, in shortened form, picosecond ultrasonics [1], [2], [3], [4]. This technique is of extensive interest because of its wide application to engineering and basic physics. Using optical interferometric techniques, one can measure ultrafast relative reflectance changes associated with acoustic echoes owing to the presence of picosecond ultrasonic pulses returning to the surface of the solid [2], [5], [6], [7], [8], [9], [10], [11]. Both the real and the imaginary parts of the relative reflectance change can be monitored, the latter proportional to the optical phase, which gives more information on the acoustic strain compared to standard transient reflectivity change measurements. Monitoring the shape of travelling strain pulses is useful in materials science, for example in buried nanostructure inspection, because the spatiotemporal profile of travelling strain pulses can be used to access stress generation mechanisms, dependent for example on electron and thermal diffusion, and on propagation processes [12], [13]. However, monitoring the shape of strain pulses is not generally possible except through the use of complicated oblique optical-incidence techniques [14], [15], because the echo shape depends on the photoelastic interaction between the light and the strain, thus mixing the effect of the optical properties of the material into the echo shape. In the case of normal incidence on isotropic materials, the strain pulse shape can be extracted only in the special case in which photoelastic effects are negligible [13]. Lai et al. proposed a reconstruction method for the strain pulse shape based on optical reflectivity changes proportional to the intensity variations of a reflected probe beam [16]. By use of a spectral sensitivity function they were able to extract the strain pulse shape from the echo shapes. However, some samples show a very weak reflectivity response, so a more robust technique sensitive to both optical amplitude and phase variations would be advantageous for this purpose. Gao et al. [17] suggested a method for reconstructing acoustic strain based on X-ray diffraction probing, but this technique requires very cumbersome apparatus and not easy to implement in a compact setting.

In this paper we introduce an analytical method to extract the travelling strain pulse profile inside an opaque solid from the transient relative reflectance change obtained by normal-incidence optical interferometry, that can monitor both optical amplitude and phase variations. This analytical method is based on understanding how the photoelastic interaction of the optical probe beam with an opaque sample with a free surface distorts the acoustic echoes and then developing a method to remove the effect of this interaction in the frequency domain [1], [18]. In brief, the real and imaginary optical reflectance changes associated with an acoustic echo are affected by the photoelastic effect, and include a damped oscillation in time [1], [3], [19]. The frequency spectrum of these optical reflectance changes show in general a peak or peaks close to the oscillation frequency. Dividing this spectrum by an appropriate filter function in frequency space, the photoelastic-dependent contributions can be removed from the frequency spectrum, and then by the use of an inverse Fourier transform the strain pulse shape can be obtained.

## 2 Theory of the echo analysis

**Fig. 1:**
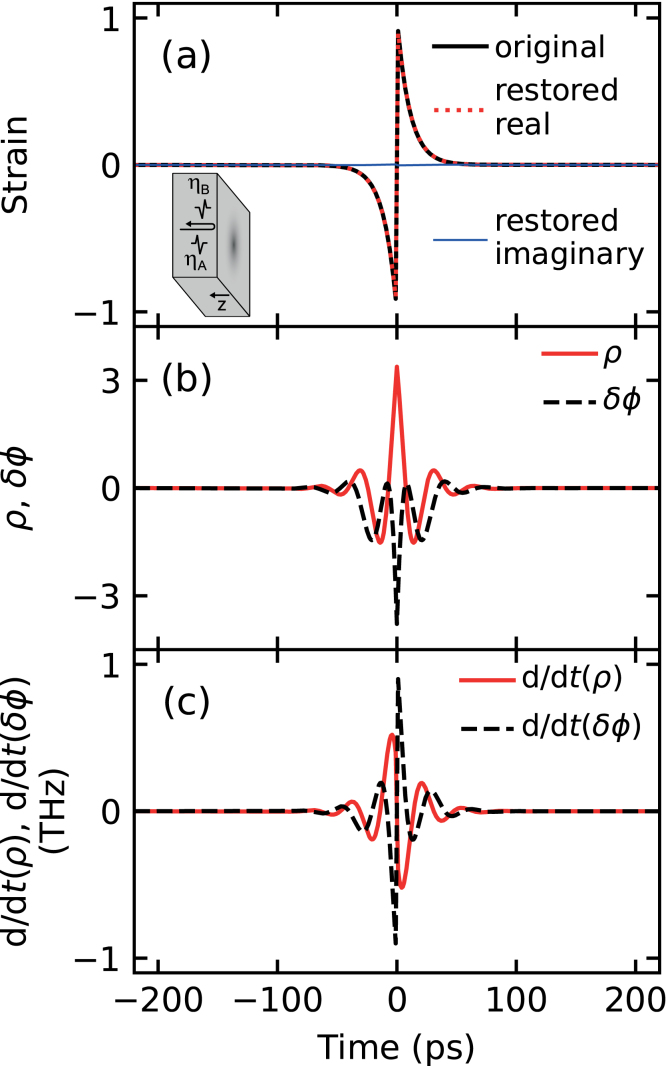
(a) Incident normalized bipolar strain pulse (black solid line) and restored strain pulse (real component: red dotted line; imaginary component: blue solid line). Inset: schematic diagram of the geometry of the strain pulse reflecting from a free surface. (b) Simulated normalized amplitude reflectance changes for this strain pulse: real (ρ, red solid line) and imaginary (δϕ, black dashed line) components vs time. (c) Temporal derivatives of the reflectance changes plotted as real (dρ/dt, red solid line) and imaginary (dδϕ/dt, black dashed line) components vs time.

We start the analysis by considering the complex reflectance change for normal optical incidence [18]. The strain pulses are considered to be of longitudinal polarization and to be unidirectional plane waves, travelling perpendicular to the surface of a semi-infinite acoustically and optically isotropic opaque material (occupying the region z>0) placed in a vacuum (or equivalently, with a stress-free boundary condition which is a good approximation for the case of contact with air). The probe light, incident from z<0, is reflected from the sample surface at z=0, and its modulated intensity is detected as a function of time by use of an optical delay line. The excitation (pump) light is modulated for the purposes of lock-in detection in order to improve the signal-to-noise ratio for echo detection. Defining the dielectric constant of the material to be $\overset{\sim }{\epsilon }$, for small modulations in reflectance the transient relative reflectance change, $\delta \overset{\sim }{r}\left(t\right)/\overset{\sim }{r}$ = ρ(t) + iδϕ(t), where ρ is the real amplitude reflectance change and δϕ is the optical phase change, and tilde ($\overset{\sim }{}$) means a complex value, can be written in the following form [18]:

(1) $$\frac{\delta \overset{\sim }{r}\left(t\right)}{\overset{\sim }{r}}=\frac{ik_{0}}{2{\overset{\sim }{a}}_{0}{\overset{\sim }{b}}_{0}}\left[\int_{0}^{\infty }{\overset{\sim }{P}}_{12}\eta \left(z^{\prime },t\right){{\overset{\sim }{a}}_{1}}^{2}\exp\left(2i{\overset{\sim }{k}}_{1}z^{\prime }\right)\text{d}z^{\prime }+{{\overset{\sim }{a}}_{1}}^{2}\left[1-\overset{\sim }{\epsilon }\right]u\left(0,t\right)\right],$$

where ${\overset{\sim }{P}}_{12}$ is the relevant photoelastic constant of the material, $\overset{\sim }{k_{i}}$ is the wave number in the region above the sample (i=0) and in the material (i=1), η(z,t) is the spatiotemporal strain profile, u(0,t) is the surface displacement at time t, and ${\overset{\sim }{a}}_{i}$, ${\overset{\sim }{b}}_{i}$ are constants proportional to the complex electric fields for the counterpropagating components of the probe light in the region above the sample (i=0) and for the unidirectionally propagating component in the material (i=1), respectively. Optical wave numbers are given by $k_{0}=2\pi /\lambda$ and ${\overset{\sim }{k}}_{1}=\overset{\sim }{n}k_{0}$, where λ is the optical wavelength in vacuum, $\overset{\sim }{n}$ = $\sqrt{\overset{\sim }{\epsilon }}$ is the complex refractive index of the material, and $\overset{\sim }{r}$ (= ${\overset{\sim }{b}}_{0}/{\overset{\sim }{a}}_{0}$) is the complex reflectance for the unperturbed material. The electric field coefficients are given by ${\overset{\sim }{a}}_{0}=k_{0}+{\overset{\sim }{k}}_{1}$, ${\overset{\sim }{b}}_{0}=k_{0}-{\overset{\sim }{k}}_{1}$, and ${\overset{\sim }{a}}_{1}=2k_{0}$, and the photoelastic constant of the material ${\overset{\sim }{P}}_{12}$ is given by ${\overset{\sim }{P}}_{12}=2\overset{\sim }{n}\text{d}\overset{\sim }{n}/\text{d}\eta$. The superposition of incident (−z propagating, $\eta_{A}\left(t+z/v\right)$) and reflected (+z propagating, $\eta_{B}\left(t-z/v\right)$) strain waves (see the inset of Fig. 1(a)) give the sum $\eta \left(z,t\right)=\eta_{A}\left(t+z/v\right)+\eta_{B}\left(t-z/v\right)$, which can be expressed as

(2) $$\eta \left(z,t\right)=\int_{-\infty }^{\infty }\left[\overset{\sim }{A}\left(\omega \right)\exp\left(-i\omega z/v\right)+\overset{\sim }{B}\left(\omega \right)\exp\left(i\omega z/v\right)\right]\exp\left(-i\omega t\right)\text{d}\omega ,$$

in terms of the strain spectra $\overset{\sim }{A}\left(\omega \right)$ and $\overset{\sim }{B}\left(\omega \right)$, which correspond to the strain propagating towards and away from the surface, respectively (see Appendix). We assume lossless, non-dispersive propagation while the strain pulse is being reflected from the free surface, i.e., q=ω/v, where q is the wave number of the strain pulse, v is a constant longitudinal sound velocity and ω is the acoustic angular frequency. In Eq. (1), u(0,t) is the +z-directed surface displacement at time t owing to the strain pulse propagation and reflection from the surface:

(3) $$u\left(0,t\right)=\int_{+\infty }^{0}\eta \left(z^{\prime },t\right)\text{d}z^{\prime }.$$

On the assumption that the surface reflects the strain pulses perfectly according to a free boundary condition, $\overset{\sim }{B}\left(\omega \right)=-\overset{\sim }{A}\left(\omega \right)$. Together with Eqs. (2), (3), the following equation can be derived from Eq. (1) (see Appendix):

(4) $$F\left[\frac{d}{dt}\frac{\delta \overset{\sim }{r}\left(t\right)}{\overset{\sim }{r}}\right]=4ik_{0}v\left[1-\frac{{\overset{\sim }{P}}_{12}}{1-{\overset{\sim }{n}}^{2}}\frac{\omega^{2}}{\omega^{2}-4{\overset{\sim }{n}}^{2}k_{0}^{2}v^{2}}\right]\overset{\sim }{A}\left(\omega \right)=\overset{\sim }{F}\left(\omega \right)\overset{\sim }{A}\left(\omega \right),$$

where F refers to a temporal Fourier transform and $\overset{\sim }{F}\left(\omega \right)$ plays the role of a filter function. In the temporal domain the derivative of the complex relative reflectance is equal to the convolution of the inverse Fourier transforms of $\overset{\sim }{F}$ and $\overset{\sim }{A}$. Eq. (4) allows $\overset{\sim }{A}\left(\omega \right)$ to be determined from the time derivative of the relative reflectance change and a knowledge of $\overset{\sim }{F}\left(\omega \right)$. By applying an inverse Fourier transform to $\overset{\sim }{A}\left(\omega \right)$, the shape of the propagating strain pulse can be calculated provided that the physical parameters used in $\overset{\sim }{F}\left(\omega \right)$ are known or derivable by fitting, i.e., $\overset{\sim }{n}$, $k_{0}$ (or λ), v and ${\overset{\sim }{P}}_{12}$ (which is complex in general).

## 3 Demonstration of the method by a simulation

To demonstrate this analytical method, we make use of a simulation of the propagation of strain pulses in the absence of ultrasonic attenuation, for the case of an opaque solid with the required stress-free boundary condition. The resultant relative reflectance changes are simulated by use of Eqs. (1), (3) for a synthetic strain pulse. For the purposes of example, we choose a bipolar strain pulse shape, $\eta_{A}\left(t\right)=\mathrm{sgn}\left(t\right)\exp\left(-v\left|t\right|/\zeta_{0}\right)$, in the form of two decaying exponential parts of opposite sign with decay constant $\zeta_{0}$ =43 nm (≈0.11λ), where the probe wavelength λ = 400 nm and the longitudinal sound velocity is v =4000 m/s. This form represents an idealized shape of a strain pulse generated thermoelastically by an ultrashort optical pulse incident at an opaque free surface of a solid in the absence of diffusion processes [1]. When such a pulse is incident on the surface, the linear one-dimensional wave equation gives the following spatiotemporal form:

(5) $$\eta \left(z\geq 0,t\right)=\mathrm{sgn}\left(t+\frac{z}{v}\right)\exp\left(-\frac{v\left|t+z/v\right|}{\zeta_{0}}\right)$$

$$\;-\mathrm{sgn}\left(t-\frac{z}{v}\right)\exp\left(-\frac{v\left|t-z/v\right|}{\zeta_{0}}\right).$$

The first term on the right-hand side represents the strain pulse $\eta_{A}\left(t+z/v\right)$ propagating from deep inside the solid towards the surface. (The solid is assumed to be much thicker than the strain pulse width and the probe beam optical penetration depth.) The centre of the strain pulse arrives at the surface (z=0) at t=0. The second term represents the inverted strain pulse $\eta_{B}\left(t+z/v\right)$, produced after reflection from the surface with a free boundary condition, which propagates away from the surface.

**Fig. 2:**
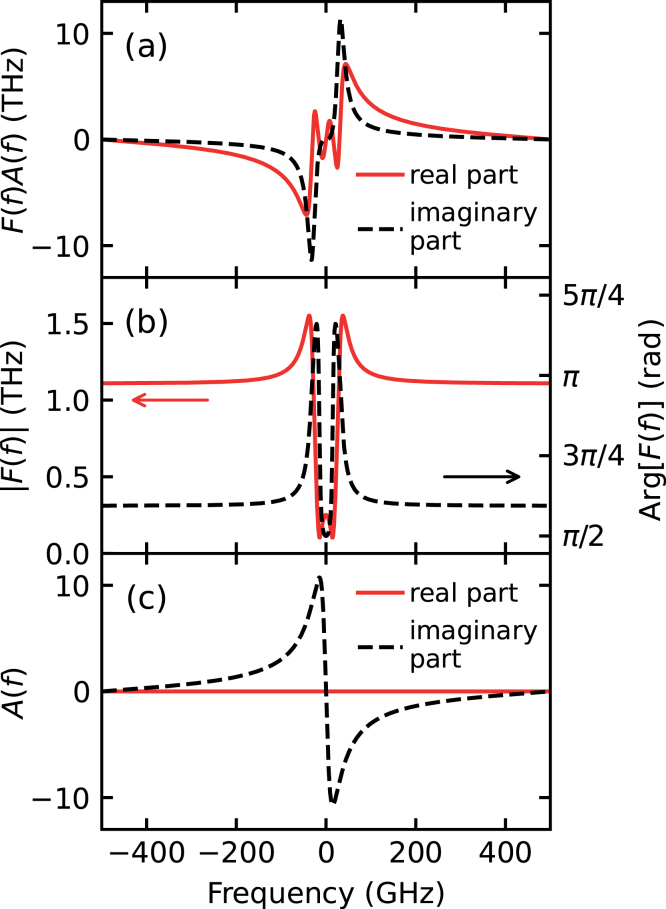
(a) Fourier transform $F\left[\left(d/dt\right)\delta \overset{\sim }{r}\left(t\right)/\overset{\sim }{r}\right]$ of the normalized temporal derivative of the reflectance change (shown as real and imaginary components in Fig. 1(c)), which can be equated to $\overset{\sim }{F}\left(f\right)\overset{\sim }{A}\left(f\right)$ (real component: red solid line; imaginary component: black dashed line). (b) Filter function modulus (red solid line) and phase (black dashed line) used for the strain restoration. (c) The spectrum $\overset{\sim }{A}\left(f\right)$ of the strain calculated from $\overset{\sim }{F}\left(f\right)\overset{\sim }{A}\left(f\right)$ (shown in (a)) divided by $\overset{\sim }{F}\left(f\right)$ (shown in (b)). Real component: red solid line; imaginary component: black dashed line.

The results of the simulation are shown in Fig. 1, Fig. 2 for probe refractive index $\overset{\sim }{n}$ =1.5+0.5i, corresponding to a probe optical absorption depth ζ=λ/4πIm$\left(\overset{\sim }{n}\right)$ ≈ 64 nm, and with the complex photoelastic constant set to $\text{d}\overset{\sim }{n}/\text{d}\eta$ = 1+1.7i.^1^ Fig. 1(a) (black solid line) shows the incident temporal strain pulse shape $\eta_{A}\left(t\right)$. Fig. 1(b) shows the simulated normalized relative reflectance changes ρ and δϕ, as calculated from Eqs. (1), (3), (5).

The echoes exhibit damped oscillations at the Brillouin period $\tau_{B}$ = λ/2nv =33.3 ps, where n = Re$\left(\overset{\sim }{n}\right)$ (with oscillation damping time ζ/v ≈ 16 ps). Fig. 1(c) shows their time derivatives (dρ/dt and dδϕ/dt) and Fig. 2(a) shows the Fourier transform $F\left[d\left(\delta \overset{\sim }{r}/\overset{\sim }{r}\right)/dt\right]$ = $\overset{\sim }{A}\left(f\right)\overset{\sim }{F}\left(f\right)$, where f is the frequency. Fig. 2(b) shows the complex filter function $\overset{\sim }{F}\left(f\right)$ calculated from Eq. (4). Fig. 2(c) shows the spectrum of the strain pulse $\overset{\sim }{A}\left(f\right)$. In this case, $\overset{\sim }{F}\left(f\right)$ shows strong variations in a similar frequency region to those found in $\overset{\sim }{A}\left(f\right)$. Fig. 1(a) also shows the restored strain pulse shape (red dotted line for real components and blue solid line for imaginary components) obtained from the inverse Fourier transform (Eq. (2)), the shape of the real component being indistinguishable from the original pulse.^2^ This demonstrates that such a filtering analysis can convert relative reflectance changes to the strain pulse shapes that produced them, provided that the relevant optical and elastic parameters are known.

**Fig. 3:**
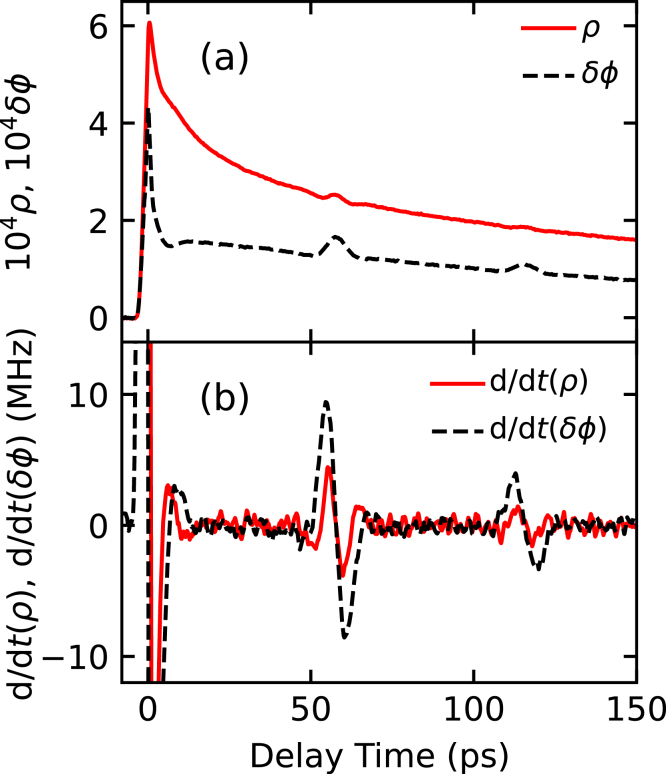
(a) Raw experimental waveforms of the relative reflectance change as a function of delay time from Ref. [20]. The red solid and black dashed lines represent ρ and δϕ, respectively. (b) The temporal derivative of the background-subtracted relative amplitude reflectance change: real (ρ, red solid line) and imaginary (δϕ, black dashed line) components.

## 4 Demonstration of the method by application to experimental results

To demonstrate the method working in practice, we apply it to the experimental results in Fig. 3(a) of Saito et al. [20], [21] for a polycrystalline Cr film of thickness 190 nm on a Si(100) substrate at a probe wavelength of 830 nm and a pump wavelength of 415 nm, with experiments conducted at normal optical incidence. Fig. 3(b) shows the values of dρ/dt and dδϕ/dt derived from the raw experimental data of Fig. 3(a) after background subtraction.^3^ The sharp response around 0 ps is caused by nonequilibrium heating and relaxation of the electron gas. Two acoustic echoes that arise from the reflection of the strain pulse from the Cr film–substrate interface are clearly distinguished near 60 and 120 ps.^4^

**Fig. 4:**
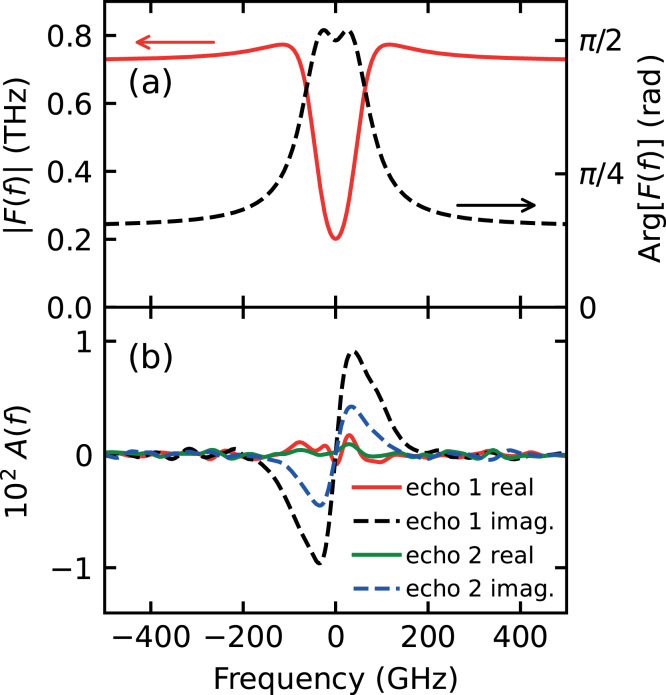
(a) Modulus (red solid line) and phase (black dashed line) of the filter function $\overset{\sim }{F}\left(f\right)$ plotted vs frequency f calculated from Eq. (4) using known physical constants and the experimental results for Cr from Ref. [21], [22]. (b) The spectrum $\overset{\sim }{A}\left(f\right)$ of the strain restored from the experimental result using $\overset{\sim }{F}\left(f\right)$. Red and green solid lines: real components of the first and second echoes, respectively; black and blue dashed lines: imaginary components of the first and second echoes, respectively.

For strain pulse shape restoration, the quantities dρ/dt and dδϕ/dt corresponding to the echoes with background subtracted are Fourier transformed. Eq. (4) yields the function $\overset{\sim }{F}\left(\omega \right)$ shown in Fig. 4(a) from the known longitudinal sound velocity v = 6650 m/s, refractive index $\overset{\sim }{n}=3.27+2.85i$ at the probe wavelength [21] and experimentally derived photoelastic constant $\text{d}\overset{\sim }{n}/\text{d}\eta$ = 5.8−4.0i [22]. Fig. 4(b) shows the real and imaginary components of the restored strain spectrum $\overset{\sim }{A}\left(\omega \right)$ for the first and second echoes, exhibiting strain components up to ∼200 GHz.

**Fig. 5:**
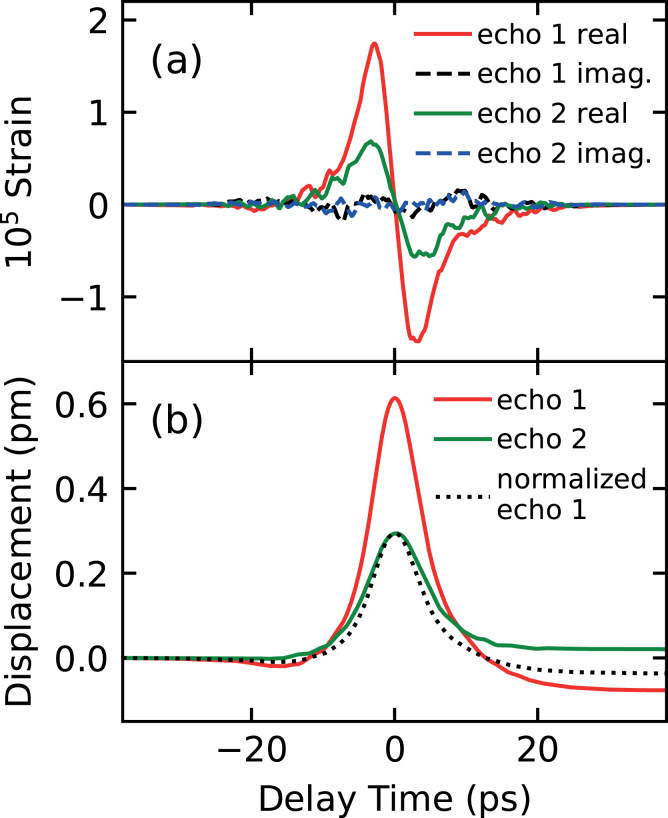
(a) Restored strain pulse shapes derived from the experimental reflectance data. Red and green solid lines: real components of the first and second echoes, respectively; black and blue dashed lines: imaginary components of the first and second echoes, respectively. (b) Transient inward surface displacements caused by the strain pulse reflection from the surface. Red and green solid lines: first and second echoes, respectively; black dotted line: normalized first echo. The time axes are chosen so that the strain pulses are centred at 0 ps.

The restored strain pulse shapes are shown in Fig. 5(a) as a function of time. The red and green solid lines correspond to the first and second echoes, respectively. The black and blue dashed lines correspond to the respective imaginary parts, which are much smaller than the real parts, being close to zero in comparison. This provides a check on the reconstruction process.

The restored strain profiles show asymmetry that originates from electron and thermal diffusion [1], [3], [23]. This is also evident in the transient inward surface displacement temporal variation arising from the strain pulse reflecting from the surface, as shown in Fig. 5(b). The red and green solid lines represent the surface displacements arising from the first and second echos, respectively. These strain pulse shapes and displacement profiles are similar to those derived from theory [20], [21]. We also plot in Fig. 5(b) the normalized first echo superimposed on the second echo, the latter showing a slightly broader shape that arises from frequency-dependent ultrasonic attenuation (∝ $f^{2}$) [21]. However, in the short time ∼10 ps, during which the strain pulse is being reflected from the free surface the lossless assumption of the theory is a reasonable approximation.

## 5 Conclusions

In conclusion, we have presented a method for analysing picosecond acoustic echoes in opaque solids for which both real and imaginary components of the reflectance changes at normal optical incidence are available. By use of a filtering analysis, one can derive the shape of the strain pulses as well as the temporal variation of the surface displacement. After demonstrating the method with synthetic strain pulse shapes, we show how to apply it to experimental data. The method requires a knowledge of the optical constants, for example obtainable by ellipsometry. The longitudinal sound velocity is also required, which can be derived from the echo arrival times provided that the thickness is known.

Extension of the method to multilayer samples in which the light penetrates into more than one layer provides a challenge for future work. Likewise for the case of semitransparent thin freestanding layers or anisotropic materials. Another interesting extension would be to the case in which the free-surface assumption no longer holds, such as when opaque solids are placed in transparent liquids. It might also be possible to directly access the derivative of the echoes experimentally by oscillating the length of the delay line and monitoring the oscillating component of the optical reflectance and phase.

## Declaration of competing interest

The authors declare that they have no known competing financial interests or personal relationships that could have appeared to influence the work reported in this paper.

## Data Availability

Data will be made available on request.

## Appendix

This Appendix provides the derivation of the filter function of Eq. (4). The Fourier transform of the strain $\eta \left(z,t\right)=\eta_{A}\left(t+z/v\right)+\eta_{B}\left(t-z/v\right)$ can be expressed as follows: (6)

$$F\left[\eta \left(z,t\right)\right]=\frac{1}{2\pi }\int_{-\infty }^{\infty }\eta_{A}\left(t+\frac{z}{v}\right)e^{i\omega t}dt+\frac{1}{2\pi }\int_{-\infty }^{\infty }\eta_{B}\left(t-\frac{z}{v}\right)e^{i\omega t}dt=\frac{1}{2\pi }\int_{-\infty }^{\infty }\eta_{A}\left(t^{\prime }\right)e^{i\omega t^{\prime }}dt^{\prime }\;e^{-i\omega z/v}+\frac{1}{2\pi }\int_{-\infty }^{\infty }\eta_{B}\left(t^{\prime }\right)e^{i\omega t^{\prime }}dt^{\prime }\;e^{i\omega z/v}=\overset{\sim }{A}\left(\omega \right)\;e^{-i\omega z/v}+\overset{\sim }{B}\left(\omega \right)\;e^{i\omega z/v}.$$

On conducting an Inverse Fourier transform, Eq. (2) is obtained.

Eq. (1) corresponds to Eq. (13) of Ref. [18]. By use of Eq. (3), Eq. (1) may be written in the form

(7) $$\frac{\delta \overset{\sim }{r}\left(t\right)}{\overset{\sim }{r}}=\frac{2ik_{0}{\overset{\sim }{P}}_{12}}{1-{\overset{\sim }{n}}^{2}}\int_{0}^{\infty }\eta \left(z^{\prime },t\right)e^{2i\overset{\sim }{n}k_{0}z^{\prime }}\text{d}z^{\prime }-2ik_{0}\int_{0}^{\infty }\eta \left(z^{\prime },t\right)z^{\prime }.$$

Its time derivative is

(8) $$\frac{d}{dt}\frac{\delta \overset{\sim }{r}\left(t\right)}{\overset{\sim }{r}}=\frac{2ik_{0}{\overset{\sim }{P}}_{12}}{1-{\overset{\sim }{n}}^{2}}\int_{0}^{\infty }\frac{\partial \eta \left(z^{\prime },t\right)}{\partial t}e^{2i\overset{\sim }{n}k_{0}z^{\prime }}\text{d}z^{\prime }-2ik_{0}\int_{0}^{\infty }\frac{\partial \eta \left(z^{\prime },t\right)}{\partial t}z^{\prime }.$$

The time derivative of Eq. (2), making use of the relation $\overset{\sim }{B}\left(\omega \right)=-\overset{\sim }{A}\left(\omega \right)$, is given by

(9) $$\frac{\partial \eta \left(z,t\right)}{\partial t}=\int_{-\infty }^{\infty }\omega \overset{\sim }{A}\left(\omega \right)e^{-i\omega t}\left(e^{i\omega z/v}-e^{-i\omega z/v}\right)d\omega .$$

For the purposes of later use, we first calculate the Fourier transform of ∂η(z,t)/∂t: (10)

$$\frac{1}{2\pi }\int_{-\infty }^{\infty }\frac{\partial \eta \left(z,t\right)}{\partial t}e^{i\omega t}dt=\int_{-\infty }^{\infty }\omega^{\prime }\overset{\sim }{A}\left(\omega^{\prime }\right)\left(e^{i\omega^{\prime }z/v}-e^{-i\omega^{\prime }z/v}\right)\delta \left(\omega -\omega^{\prime }\right)d\omega^{\prime }=\omega \overset{\sim }{A}\left(\omega \right)\left(e^{i\omega z/v}-e^{-i\omega z/v}\right).$$

We have made use of the relation $\frac{1}{2\pi }\int_{-\infty }^{\infty }e^{i\left(\omega -\omega^{\prime }\right)t}dt=\delta \left(\omega -\omega^{\prime }\right)$.

The Fourier transform of the time derivative of the amplitude reflectance change is given by (11)

$$F\left[\frac{d}{dt}\frac{\delta \overset{\sim }{r}\left(t\right)}{\overset{\sim }{r}}\right]=\frac{1}{2\pi }\int_{-\infty }^{\infty }\frac{d}{dt}\frac{\delta \overset{\sim }{r}\left(t\right)}{\overset{\sim }{r}}e^{i\omega t}dt=4ik_{0}v\left[1-\frac{{\overset{\sim }{P}}_{12}}{1-{\overset{\sim }{n}}^{2}}\frac{\omega^{2}}{\omega^{2}-4{\overset{\sim }{n}}^{2}k_{0}^{2}v^{2}}\right]\overset{\sim }{A}\left(\omega \right).$$

Integration with respect to spatial coordinate $z^{\prime }$ requires the assumption that the acoustic field should be zero at z=+∞. This completes the proof of Eq. (4). An alternative form of $\overset{\sim }{F}\left(f\right)$ is

(12) $$\overset{\sim }{F}\left(f\right)=\frac{8\pi iv}{\lambda }\left[1-\frac{{\overset{\sim }{P}}_{12}}{1-{\overset{\sim }{n}}^{2}}\frac{f^{2}}{f^{2}-4{\overset{\sim }{n}}^{2}v^{2}/\lambda^{2}}\right].$$

It is possible to carry out the strain restoration process without taking the time derivative of $\delta \overset{\sim }{r}\left(t\right)/\overset{\sim }{r}$ by use of a similar filter function definition, but this results in a discontinuity in the filter function at zero frequency and to a stronger variation in this function around this frequency, requiring the use of smaller frequency steps and a longer time range. In addition, the filter function tends to zero at high frequencies, thus requiring more care in the restoration process to avoid the introduction of noise. We have therefore adopted an approach based on $\left(d/dt\right)\delta \overset{\sim }{r}\left(t\right)/\overset{\sim }{r}$.

## Footnotes

1 The time step of the data is 1 ps over a range of −500 to 500 ps. The spatial step is 0.1 nm over a range of 2400 nm in depth for use in Eq. (1). The frequency step is 1 GHz and the Nyquist frequency, 0.5 THz, is high compared to the frequencies in the strain pulse, thus avoiding perturbations to the restoration process. Zero padding is not used.

2 The discrepancy is ∼$10^{-5}$ for our chosen parameters. The error arises from the integral calculations of the amplitude reflectance, defined in Eq. (1), for the original bipolar strain pulse used rather than from the forward and inverse Fourier transforms defined in Eqs. (2), (4). This error is in general much smaller than the experimental noise or wave-propagation simulation errors.

3 The background variation is determined by fitting to a time-decaying exponential function. Time derivatives are done after smoothing with a Savitzky–Golay filter using a 180-point window-width corresponding to a temporal duration of 2.4 ps and a polynomial of order 1.

4 The time step of the experimental data is 13.3 fs. 5000 points corresponding to 67 ps around each echo are selected for the analysis. Zero padding is also carried out to reduce the frequency step to 3 GHz. The Nyquist frequency, 37.5 THz, is high compared to the frequencies in the strain pulse, thus avoiding perturbations to the restoration process.
